# Mental Health Impact among Survivors from COVID 19 Pneumonia, Almoosa Hospital Experience

**DOI:** 10.1192/j.eurpsy.2022.1285

**Published:** 2022-09-01

**Authors:** S. Osman, S. Alkhalifa

**Affiliations:** Almoosa Specialsts Hispital, Psychiatry, alahsa, Saudi Arabia

## Abstract

**Introduction:**

COVID-19 is associated with mental manifestations, Anxiety and depression appear to be common amongst people hospitalized for COVID-19.

**Objectives:**

evaluate the emotional stress resulting from infection and assess its impact on the mental health of patients who recovered from COVID-19 pneumonia.

**Methods:**

It is a cross-sectional study, The mental health assessment tool DASS2 (Arabic version) was applied in collecting the data for the study. Demographic characteristics, chronic disease status, COVID 19 pneumonia, oxygen saturation level were recorded at the follow-up visit, soon after the psychiatric evaluation. Psychological distress was assessed An Arabic version of the Depression, Anxiety, and Stress Scale-21 (DASS-21) was used to assess the mental health status. **Statistical analysis by** (SPSS, version 25).

**Results:**

466 patients were consented prior to enrollment in the study, out of the total respondents; (53.2%) were females, anxiety rate was found in (18%), stress in (17%), and depression in (14%) of the patients, significantly elevated blood levels of the inflammatory marker in patients with depression and anxiety, increase in the rates of depression with male gender, increasing age and longer duration of ICU stay respectively, with non-significant p-values. There was also a small increase in the period stayed in ICU among those who developed depression and anxiety. Reduced oxygen saturation in COVID-19 patients with depression was 4 times those with no depression.

**Conclusions:**

prolonged ICU stays and reduced oxygen saturation was associated with a high rate of depression in patients with COVID-19, as well as elevated levels of the inflammatory marker D-dimer with depression and anxiety.

**Disclosure:**

No significant relationships.

